# Evaluating the Effectiveness of Generative AI for the Creation of Patient Education Materials on Coronary Heart Disease: A Comparative Study

**DOI:** 10.2196/78816

**Published:** 2025-11-21

**Authors:** Xiaofang Jiang, Jingbang Liu, Shanshan Dai, Xiawen Mao, Rongping Cha, Lili Wu

**Affiliations:** 1 Nursing Department, Sir Run Run Shaw Hospital, Zhejiang University School of Medicine, 3 East Qingchun Road, Hangzhou, Zhejiang Province, 310000, China, 86 13777392684

**Keywords:** generative artificial intelligence, large language models, coronary heart disease, patient education, ChatGPT, DeepSeek

## Abstract

**Background:**

Generative artificial intelligence (AI) has shown great potential in various fields, including health care. However, its application for developing patient education materials (PEMs), particularly for those with coronary heart disease (CHD), remains underexplored. Traditional methods for creating these materials are time-consuming and lack personalization, which limit their effectiveness.

**Objective:**

This study aims to explore the effectiveness of generative AI tools (ChatGPT and DeepSeek) at generating PEMs for patients with CHD and to compare them with materials developed by a professional medical team.

**Methods:**

In February 2025, PEMs for patients with CHD were developed using a framework designed by a professional medical team. Structured prompts were used to generate materials through 2 generative AI models—ChatGPT-4o and DeepSeek R1. These AI-generated materials were compared with those created by the medical team in terms of development time, readability, understandability, actionability, and accuracy.

**Results:**

The total time for manual preparation was 14 hours, while ChatGPT and DeepSeek consumed 0.62 hours and 0.78 hours, respectively. Regarding readability, the frequency of difficult words was more variable in manually written and ChatGPT materials, while DeepSeek showed more consistency. The proportion of simple sentences was highest with DeepSeek, followed by ChatGPT, with complete separation between manually written and ChatGPT (*δ*=1). Content word frequency was highest in manually written PEMs, while ChatGPT had the lowest but most stable values. Personal pronouns were most frequently used in manually written PEMs, with high variability, and least used in DeepSeek, which was stable. All 3 methods had similar readability levels and reached Chinese elementary school–level readability for the proportions of simple sentences and personal pronouns, with high school–level difficulty of words and content word frequency. The understandability and actionability scores were above 70, with ChatGPT being more stable for understandability and DeepSeek being more stable for actionability. No significant differences were found between groups. In terms of accuracy, intergroup comparisons showed significant differences (*H*=7.27, *P*=.03) but no significant differences in multiple comparisons. The direct comparison between ChatGPT and DeepSeek showed a negligible effect size (*δ*=0.02), with no significant difference (*z*-score=−0.06, *P*=.96). Accuracy issues in the AI-generated materials were noted by 4 of 8 experts.

**Conclusions:**

Generative AI significantly improved the efficiency of developing PEMs for patients with CHD. The materials generated by ChatGPT-4o and DeepSeek R1 were comparable to the professionally written ones in terms of readability, understandability, and actionability. However, improvements related to reducing the number of difficult words and increasing content word frequency are needed to enhance readability. The accuracy of AI-generated materials still poses concerns, including potential AI “hallucinations,” and requires review by health care professionals. Generative AI holds considerable potential for generating PEMs, and future research should assess its applicability and effectiveness in real-world patient and family contexts.

## Introduction

Coronary heart disease (CHD), or coronary atherosclerotic heart disease, remains one of the leading causes of morbidity and mortality worldwide [1]. According to the 2019 Global Burden of Disease study, approximately 17.9 million people worldwide died from cardiovascular diseases in 2019, accounting for 32% of all global deaths, demonstrating the continued rise in the global burden of cardiovascular diseases [2]. Although revascularization techniques have significantly improved survival rates, the health literacy of patients with CHD remains relatively low, posing a significant barrier to effective disease management and patient empowerment [3]. The key to preventing cardiovascular diseases lies in reducing controllable risk factors. Public health strategies include reducing risk factors at the population level, as well as implementing primary and secondary prevention and treatment at the individual level [4]. Health education is a vital component for improving patient outcomes by increasing compliance with treatment regimens, enhancing self-management, and fostering informed decision-making [5]. However, traditional methods of developing patient education materials (PEMs) rely heavily on manual processes [6-8], which are time-consuming and require substantial input from health care providers [9,10]. These manual materials are often generic and may lack personalization, making it difficult to tailor information to the specific needs of individual patients. Additionally, the materials may not reflect the latest scientific evidence or guidelines due to a lack of rapid updating, which could hinder the dissemination of up-to-date medical information to patients.

Generative artificial intelligence (AI) has emerged as a promising solution to address these pain points. Powered by advancements in machine learning and large language models, generative AI enables the rapid creation of content that can be tailored to specific patient needs [11]. In health care, generative AI has shown potential in a range of applications, such as generating medical records, enhancing clinical decision support systems, and supporting medical education [12-14]. Certain applications have also been implemented for providing medical consultation and education; have been used to generate or optimize the PEMs for diseases in departments such as otolaryngology, dermatology, and gastroenterology; and have been preliminarily validated [8-10,13].

The release of DeepSeek R1 in January 2025 accelerated the adoption of generative AI tools [15]. Some international studies have examined and validated DeepSeek’s effectiveness at supporting public cardiopulmonary resuscitation training [16]. Nevertheless, research using DeepSeek for the development of Chinese-language PEMs remains scarce. Some studies have evaluated various generative AI models, particularly ChatGPT, for generating PEMs [17,18]. Compared with ChatGPT, the capabilities of DeepSeek have not yet been widely explored in the context of PEMs, especially for chronic diseases such as CHD. In-depth comparisons and evaluations of generative AI tools are still lacking in this domain. Generative AI technologies can algorithmically generate logically structured and instructive content in a short period of time, offering new opportunities to enhance the efficiency and innovation of PEM development. However, issues such as hallucinations and biases may lead to misinformation, posing risks and challenges to patient safety and medical ethics [15,19].

This study seeks to fill this gap by comparing the quality of PEMs for patients with CHD generated by 2 generative AI tools: ChatGPT-4o and DeepSeek R1. ChatGPT-4o is based on the GPT-4 architecture and was pretrained on a large corpus of open-text data including news articles, books, and online content. It demonstrates strong generative capabilities across a wide range of language tasks, but it may exhibit certain hallucination phenomena when generating information [20]. DeepSeek R1, a deep learning model with reasoning capabilities, offers unique advantages for logical reasoning and content generation in professional fields. Compared with ChatGPT-4o, DeepSeek R1 is more focused on handling tasks that require deep logical analysis [21]. However, due to its more complex reasoning process, DeepSeek R1 tends to have a slower generation speed. These 2 models, based on different technical architectures and training strategies, have distinct strengths and limitations. This study aimed to compare the quality of CHD-related PEMs generated by these 2 generative AI tools with those developed by a professional health care team, providing empirical evidence to inform the application and promotion of generative AI in PEM development.

## Methods

### Overview

This project is a comparative study, with samples consisting of 3 sets of PEMs for CHD: 1 set manually written by a professional health care team and 2 sets generated by ChatGPT-4o (ChatGPT) and DeepSeek R1 (DeepSeek). The quality differences between the 3 groups were evaluated by assessing the preparation time, readability, understandability, actionability, and accuracy of the PEMs.

Each material set consisted of 3 PEMs routinely developed by the cardiology health education team, namely “Coronary Heart Disease Knowledge,” “Coronary Artery Minimally Invasive Interventional Therapy,” and “Common Questions About Coronary Artery Minimally Invasive Interventional Therapy.” This study did not involve direct participation of patients, and patient-related information or data were not included.

### Method for Developing Manually Written PEMs

Due to the absence of an authoritative official website in China for downloading standardized PEMs, each hospital develops PEMs according to its specific needs. In our hospital, the PEMs were developed following the hospital’s established process for PEMs [22]. The development team consisted of experienced cardiologists, a nurse educator, and health educators. Initially, the team conducted a needs assessment and followed the hospital’s guidelines for PEM development. The materials were then drafted based on a review of relevant literature [23-25] and discussions within the group. Afterward, the draft was submitted to the hospital’s health promotion nurse for review, who provided feedback on the content and structure. The revised draft was then shared with 10 patients or family members for further feedback. After collecting their suggestions, the draft was revised again and ultimately reviewed and finalized by senior cardiology specialists with high-level professional titles. The final versions of the 3 materials on CHD education are listed in Table 1.

**Table 1.** List of 3 patient education materials (PEMs) for coronary heart disease (CHD).

PEM name	Contents
Coronary Heart Disease Knowledge	What is CHD? What is the current status of CHD? What are its dangers? What are the risk factors for CHD? How do atherosclerotic plaques form? Does CHD always involve chest pain? What are the symptoms of CHD? What are the current treatment options for CHD?
Coronary Artery Minimally Invasive Interventional Therapy	What is coronary angiography (CAG)? What is percutaneous coronary intervention (PCI)? What are the types of stents? Who needs coronary artery intervention therapy? What preoperative tests are needed before coronary intervention therapy? What preparations are needed before coronary intervention therapy? What are the specific steps of coronary artery intervention therapy? What sensations will you feel during the procedure? What should you pay attention to after coronary intervention therapy? What should you do after discharge?
Common Questions About Coronary Artery Minimally Invasive Interventional Therapy	Is everything fine after coronary intervention therapy? Can the stent fall off? Can patients resume normal work and life after coronary intervention therapy? If I feel fine, can I stop taking antiplatelet drugs? Can I undergo MRI^a^ or CT^b^ scans after coronary artery stent implantation? What should I pay attention to when traveling after coronary intervention therapy? What should be done in the case of a heart attack?

a MRI: magnetic resonance imaging.

b CT: computed tomography.

### Method for Developing AI-Generated PEMs

All PEMs generated by ChatGPT and DeepSeek were created on February 17, 2025. To ensure the accuracy and relevance of the generated content, a nursing expert with an Artificial Intelligence Generated Content Mentor-level certificate from the Ministry of Industry and Information Technology, along with a health promotion nurse in health education, collaboratively developed the structured prompts [26] and optimized the prompts through follow-up questions, examples, and other methods. The prompts (see Multimedia Appendix 1) were adapted from previous studies based on a certain structure and further optimized and adjusted by our team before being used to generate the final content, to ensure their effectiveness [27].

The structured prompts included key components such as role setting, content background, specific tasks, and detailed requirements. The structured elements covered the themes, target audience, objectives, and a manual table of contents that aligned with the structure of the materials manually created by the health education team. Additionally, the prompts included the necessary background knowledge, and restrictions were applied to the language style in order to enhance readability and ensure that the language was accessible to the target population. Once the prompts were finalized and optimized, they were input into the 2 generative AI models with the file “List of Three PEMs for CHD.” Both models independently generated the complete set of PEMs for patients with CHD, and the set of PEMs was then compiled into a finalized format. We did not make any modifications to the final content. The results generated during the prompt optimization process, due to their low quality, were discarded, and only the final version was retained for comparison.

### Evaluation Metrics

The evaluation criteria included 5 aspects: time spent, readability, understandability, actionability, and accuracy of PEMs for CHD.

#### Time Spent

The time spent developing manually written PEMs (measured in hours) included the time spent on needs assessment by the specialized team, literature review, group discussions, and the drafting of the initial materials. It also included the time for the health promotion nurse to review the materials and provide feedback; the time for 10 patients or family members to read and provide feedback, followed by revisions based on their input; and finally, the time spent by senior specialists in the field for the final review and revision of the materials. The time was collected by the cardiology health educator.

As for AI-generated PEMs, the researchers used a timer to record the total time (in minutes) from the start to the completion of each set of PEMs. This included the time for writing the structured prompts, optimizing and modifying the prompts, generating and refining the content, and organizing the materials into their final format. The time was also collected by the cardiology health educator.

#### Readability

Text readability refers to the difficulty or ease with which a text can be read and understood. It is an important linguistic characteristic of text and is widely used in fields such as information science, journalism, and health education [28,29]. The Chinese Readability Indicator and Evaluator (CRIE) system is an automated tool for analyzing the readability of Chinese texts [30,31]. It primarily analyzes 4 core linguistic features: the number of difficult words, the proportion of simple sentences, the logarithmic value of content word frequency, and the number of personal pronouns.

Chinese and alphabetic languages have significant morphological differences. In Chinese, characters are formed by combining strokes and components, and words are made up of individual characters, each of which is monosyllabic. Therefore, word length metrics commonly used in alphabetic languages, such as the number of letters or syllables, are completely ineffective when analyzing Chinese. CRIE defines words not included in the common 3000 high-frequency Chinese word list as difficult words. Difficult words reflect the complexity of the text’s vocabulary: the more difficult words there are, the higher the reading difficulty and the lower the readability.

A simple sentence refers to a sentence without complex subordinate clauses. The proportion of simple sentences refers to the percentage of simple sentences in the text, calculated as the number of simple sentences divided by the total number of sentences. A higher proportion of simple sentences indicates lower reading difficulty and higher readability.

The logarithmic value of content word frequency measures the average frequency of content words (including nouns, verbs, and adjectives) in the text. A larger logarithmic value indicates a higher average frequency of content words, meaning these words are repeated more often, making them more familiar to readers, reducing cognitive load, and increasing text readability. Conversely, a lower content word frequency suggests the use of a broader vocabulary, requiring readers to constantly adapt to new content words, which increases cognitive load and decreases readability. For example, in the case of coronary heart disease, the term ”risk factors“ would indicate a high content word frequency if used consistently throughout the text, suggesting lower cognitive load and higher readability. However, if synonyms like ”harmful factors“ or ”high-risk factors” are continuously introduced, it would indicate a lower content word frequency, greater cognitive load, and lower readability.

The number of personal pronouns refers to the frequency of personal pronouns in the text. Excessive use of personal pronouns may affect the coherence of the text and the clarity of references, increasing the reading difficulty and decreasing readability.

The 3 sets of PEMs for patients with CHD were analyzed using this system, comparing the 4 core linguistic features across the materials. The analysis assessed the readability of the 3 sets of materials and their corresponding grade levels. The system demonstrated accuracy rates of 93% for word analysis and 86% for syntactic analysis, and it demonstrated grade classification accuracy rates of 72.92% for primary and secondary school textbooks, 75% for second language texts, and 80.83% for natural science texts.

#### Understandability and Actionability

In this study, the Patient Education Materials Assessment Tool for Printable Materials (PEMAT-P) was used to evaluate the effectiveness and applicability of PEMs. This tool was developed by the Agency for Healthcare Research and Quality in 2012 [32]. Compared with earlier tools, PEMAT-P is designed to be more user-friendly [33,34]. The Chinese version of PEMAT-P was adapted by Yu et al in 2023 [35]. The adapted tool includes 2 subscales—understandability and actionability—with a total of 7 dimensions and 23 items. Specifically, 16 items assess understandability, and 7 items evaluate actionability. Response options include “Agree” (1 point), “Disagree” (0 points), and “Not Applicable (N/A),” with “Not Applicable” items excluded from the final score. The total score ranges from 0 to 100, with higher scores indicating better understandability or actionability. A score greater than 70 indicates that the material has good understandability or actionability. The formula for calculating the Chinese version of PEMAT-P is as follows: PEMAT-P score = (total score/total number of items) × 100. The scale demonstrated high reliability, with a scale content validity index of 0.970, a Kaiser-Meyer-Olkin value of 0.655, and a Bartlett test of sphericity *χ*^2^ value of 710.200 (*P*<.001). The overall Cronbach α coefficient was 0.754, with Cronbach α for the understandability subscale at 0.802 and for the actionability subscale at 0.615.

We invited 8 senior experts in health education and cardiology to assess the materials using the Chinese version of the PEMAT-P. The 8 experts had a mean age of 45.00 (SD 4.41) years. Among them, 4 held senior professional titles, 3 had associate senior titles, and 1 had an intermediate title. Their educational backgrounds included 2 with doctorates, 3 with master’s degrees, and 3 with bachelor’s degrees. The group included 2 cardiologists, 3 cardiology nurses, and 3 health education experts (including 2 from public health and 1 from a hospital). The mean length of professional experience of the experts was 16.25 (SD 7.38) years. Of the experts, 5 were listed in the provincial-level health education expert database. The 3 sets of PEMs were randomly coded by the researchers and sent to the experts via email, along with the expert evaluation letter, the expert background and authority questionnaire, the PEMAT-P and its user manual, and the 3 sets of PEMs for CHD. Experts were informed to return the results to the researchers’ emails within 1 week. The evaluation letter included the researchers’ emails and phone numbers, and the experts were advised that they could contact the researchers if they had any questions. The response rate for the expert inquiry was 100%, with an expert authority coefficient of 0.877.

#### Accuracy

Accuracy is one of the key indicators used to assess the quality of PEMs [36]. In this study, a Likert 6-point scale was used to assess accuracy [37], where a score of 1 represented completely incorrect, 2 indicated more incorrect than correct, 3 meant equal elements of correct and incorrect, 4 represented more correct than incorrect, 5 indicated almost entirely correct, and 6 signified completely correct. The accuracy of the 3 sets of PEMs for patients with CHD was evaluated by 8 experts. The experts were asked to provide written descriptions in the comment section if they identified any specific accuracy issues with the materials. The response rate for the expert inquiry was 100%.

### Statistical Methods

Data entry and verification were conducted by 2 trained team members using Excel 2021 (Microsoft Corp). Statistical analysis was performed using SPSS version 25 (IBM Corp) and R 4.5.1. The Shapiro-Wilk test was used to assess data normality, and the Levene test was used to check the homogeneity of variances. Due to the very small sample size, readability analysis is presented descriptively using median, minimum, and maximum values; strip plots; and box plots. Cliff delta effect size *δ* and its 95% CI were used for comparisons between 2 groups. Understandability and actionability were described using means and SDs, with intergroup comparisons conducted using the *F* test. Multiple comparisons were made using the least significant difference test, and the comparisons between ChatGPT and DeepSeek were performed using *t* tests. Effect sizes were calculated using Cohen *d*, corrected with Hedges *g*. For accuracy analysis, due to non-normal distribution of the data, median and interquartile ranges were used. Intergroup comparisons were conducted using the Wilcoxon rank-sum test, multiple comparisons were made using the Kruskal-Wallis 1-way ANOVA test (for k samples), and the comparison between ChatGPT and DeepSeek was conducted using the Wilcoxon rank-sum test. Effect sizes are presented using δ and its 95% CI.

### Ethical Considerations

The local institutional review board adheres to the Declaration of Helsinki. After consultation with the board, it was determined that no formal ethical approval was required for this study, as no human or animal participants were involved. The study was designed to ensure ethical standards were maintained, and after providing informed consent, all professionals participated in the evaluation voluntarily and were not compensated. We implemented strict confidentiality measures, and access to the data was only provided to the research team.

## Results

### Comparison of Development Time

The specific development times for manually written PEMs were as follows: 1 hour for the needs assessment by the specialized team, 2 hours for literature review, 1 hour for group discussions, and 3 hours for drafting the initial materials, totaling 7 hours. The time for the hospital’s health promotion nurse to review and provide feedback was 0.5 hours, followed by 6 hours for 10 patients or family members to read the materials and provide feedback for revisions. Finally, 0.5 hours were spent by senior specialists to review and revise the materials, bringing the total development time for the control group to 14 hours.

As for AI-generated PEMs, the time for writing and optimizing the prompts was 15 minutes. The time for using ChatGPT to generate and optimize the materials was 17 minutes, and 5 minutes were used to organize the final version. For DeepSeek, the time for generating and optimizing the materials was 25 minutes, and 7 minutes were spent on organization. The total time for generating materials using ChatGPT was 0.62 hours, and for DeepSeek, it was 0.78 hours, with an average time of 0.7 hours. The time spent on manual development was 20 times longer than the time required by generative AI.

### Comparison of Readability

For the number of difficult words, both the manually written and ChatGPT materials showed extremely high values (737 and 740, respectively), resulting in very long whiskers in the box plots and indicating high variability. This suggests large fluctuations in the use of difficult words. In contrast, DeepSeek data were more concentrated and stable, with no outliers, indicating tighter control over vocabulary difficulty. The Cliff delta effect size *δ* between ChatGPT and DeepSeek indicated a medium effect; however, the 95% CI included 0, suggesting high uncertainty in the difference due to the small sample size.

Regarding the proportion of simple sentences, DeepSeek was higher overall than the other 2 groups, with a tightly clustered distribution and the highest proportion of simple sentences, suggesting a tendency to enhance readability through simpler sentence structures. ChatGPT had the lowest proportion, indicating more complex sentence construction. The proportion of simple sentences in the manually written materials was intermediate and showed a tight distribution. The data between manually written and ChatGPT materials were completely separated, while the 95% CI for *δ* between manually written and DeepSeek materials included 0, indicating high uncertainty. A large effect size was observed between ChatGPT and DeepSeek.

For content word frequency, manually written materials had the highest value but with an extreme outlier (1.253), suggesting the highest repetition of content words but also instability. ChatGPT had the lowest and most stable values, while DeepSeek was moderate and stable. This indicates that generative AI models—particularly ChatGPT—tended to use a wider variety of content words, which may increase cognitive load and reduce readability. All pairwise comparisons among the 3 groups showed complete separation.

In terms of personal pronoun usage, manually written materials used the most, with high variability—possibly due to narrative needs. DeepSeek used the fewest and was the most stable, while ChatGPT was intermediate. This suggests that generative AI models, especially DeepSeek, tend to use fewer personal pronouns to improve readability. However, the 95% CIs of *δ* for all pairwise comparisons included 0, indicating high uncertainty.

**Figure 1.**
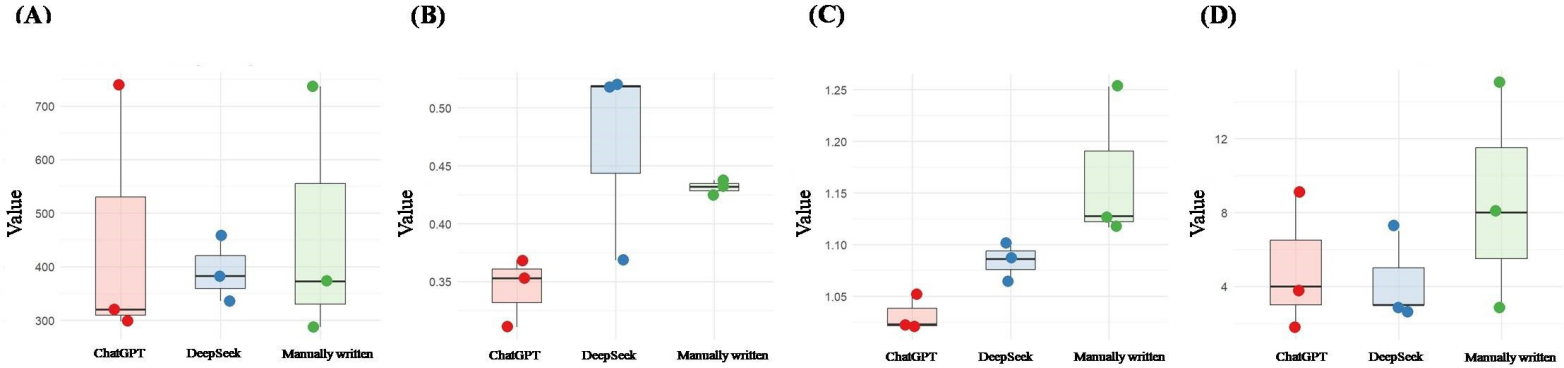
Strip plots and box plots of the 3 methods to generate patient education materials (PEMs) for coronary heart disease (CHD): (A) number of difficult words, (B) proportion of simple sentences, (C) logarithmic value of content word frequency, (D) number of personal pronouns.

Overall, DeepSeek demonstrated stability in controlling difficult words, simple sentence proportions, and personal pronoun usage. ChatGPT had a lower proportion of simple sentences, more complex syntax, and the lowest content word frequency, reflecting more diverse vocabulary usage. Manually written materials had the highest content word frequency and lowest cognitive load but showed high variability and risk of outliers. See Figure 1 and Table 2.

**Table 2.** Comparison of readability scores among the 3 methods of generating patient education materials (PEMs) for coronary heart disease (CHD).

Readability	Manually written		ChatGPT		DeepSeek		Manually written versus ChatGPT, δ (95% CI)	Manually written versus DeepSeek, δ (95% CI)	ChatGPT versus DeepSeek, δ (95% CI)
	Median (range)	Mean	Median (range)	Mean	Median (range)	Mean			
Difficult words, n	373 (287-737)	465.67	320 (298-740)	452.67	383 (336-458)	392.33	−0.11 (−0.79 to 0.69)	−0.11 (−0.88 to 0.82)	−0.33 (−0.95 to 0.81)
Proportion of simple sentences	0.43 (0.43-0.44)	0.43	0.35 (0.31-0.37)	0.34	0.52 (0.37-0.52)	0.47	1.00 (0.14 to 1.00)	−0.33 (−0.91 to 0.68)	−0.89 (−0.99 to –0.23)
Content words, n	1.13 (1.12-1.25)	1.17	1.02 (1.02-1.05)	1.03	1.09 (1.07-1.10)	1.08	1.00 (0.14 to 1.00)	1.00 (0.14 to 1.00)	−1.00 (−1.00 to −0.14)
Personal pronouns, n	8 (3-15)	8.67	4 (2-9)	5.00	3 (3-7)	4.33	0.33 (−0.68 to 0.91)	0.56 (−0.62 to 0.96)	0.11 (−0.82 to 0.88)

The proportions of simple sentences and personal pronoun usage across all 3 PEM groups aligned with the reading level of Chinese elementary school students. However, both the difficult word frequency and content word frequency corresponded to a high school reading level, exceeding the commonly recommended 6th-grade level for PEMs, indicating that neither manually written nor AI-generated PEMs met the target readability level in terms of vocabulary complexity.

### Comparison of Understandability and Actionability

The mean scores for understandability and actionability of all 3 sets of PEMs for CHD were higher than 70, indicating that both the manually written and AI-generated PEMs demonstrated good levels of understandability and actionability.

In terms of understandability, the SDs were relatively large for the “Coronary Heart Disease Knowledge“ section in the manually written materials and for the “Coronary Artery Minimally Invasive Interventional Therapy” and “Common Questions About Coronary Artery Minimally Invasive Interventional Therapy” sections in the DeepSeek-generated materials, indicating a higher degree of data dispersion.

**Figure 2.**
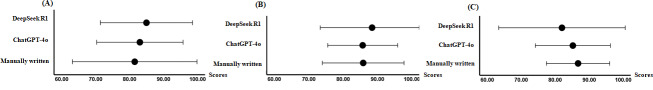
Mean (SD) understandability scores for the 3 methods of generating patient education materials (PEMs) for coronary heart disease (CHD) for each of the 3 PEM sections: (A) Coronary Heart Disease Knowledge, (B) Coronary Artery Minimally Invasive Interventional Therapy, (C) Common Questions About Coronary Artery Minimally Invasive Interventional Therapy.

For actionability, higher SDs were observed in the “Coronary Heart Disease Knowledge” and “Common Questions About Coronary Artery Minimally Invasive Interventional Therapy” sections in the manually written materials, as well as in the “Coronary Heart Disease Knowledge” and “Coronary Artery Minimally Invasive Interventional Therapy” sections generated by ChatGPT. These findings suggest that ChatGPT was more stable in understandability, while DeepSeek showed greater stability in actionability (see Figures2  3). Intergroup and multiple comparisons among the 3 PEMs showed no statistically significant differences in understandability nor actionability. Direct comparisons between PEMs generated by ChatGPT and DeepSeek indicated no statistically significant differences.

**Figure 3.**
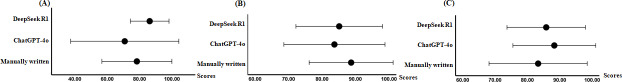
Mean (SD) actionability scores for the 3 methods of generating patient education materials (PEMs) for coronary heart disease (CHD) for each of the 3 PEM sections: (A) Coronary Heart Disease Knowledge, (B) Coronary Artery Minimally Invasive Interventional Therapy, (C) Common Questions About Coronary Artery Minimally Invasive Interventional Therapy.

These findings suggest that the understandability and actionability levels of CHD PEMs developed using the 3 methods are comparable and that the quality of PEMs generated by the 2 generative AI models is also equivalent in these dimensions (see Table 3).

**Table 3.** Comparison of understandability and actionability scores of the 3 methods of generating patient education materials (PEMs) for coronary heart disease (CHD), as evaluated by 8 experts.

PEM section	Manually written, mean (SD)	ChatGPT, mean (SD)	DeepSeek, mean (SD)	*F* (*df*)^a^	*P* value^a^	Hedges *g*^b^ (95% CI)	*t*^b^ (*df*)	*P^b^* value
Understandability								
Coronary Heart Disease Knowledge	81.52 (18.33)	83.04 (12.71)	84.98 (13.56)	0.11 (2,21)	.90	−0.14 (−1.15 to 0.88)	−0.30 (14)	.77
Coronary Artery Minimally Invasive Interventional Therapy	85.84 (11.88)	85.69 (10.16)	88.42 (15.07)	0.12 (2,21)	.89	−0.20 (−1.22 to 0.82)	−0.43 (14)	.68
Common Questions About Coronary Artery Minimally Invasive Interventional Therapy	86.73 (9.30)	85.19 (11.03)	81.99 (18.60)	0.25 (2,21)	.78	0.20 (−0.82 to 1.21)	0.42 (14)	.68
Actionability								
Coronary Heart Disease Knowledge	78.30 (21.63)	70.80 (33.43)	86.22 (11.95)	0.83 (2,21)	.45	−0.58 (−1.62 to 0.45)	−1.23 (14)	.24
Coronary Artery Minimally Invasive Interventional Therapy	88.72 (12.54)	83.72 (15.11)	85.15 (12.93)	0.29 (2,21)	.75	−0.10 (−1.11 to 0.92)	−0.20 (14)	.84
Common Questions About Coronary Artery Minimally Invasive Interventional Therapy	83.30 (15.17)	88.30 (12.80)	85.80 (12.12)	0.28 (2,21)	.76	0.19 (−0.83 to 1.21)	0.40 (14)	.69

a Comparisons among the 3 groups.

b Direct comparison between ChatGPT and DeepSeek.

### Accuracy Results

The accuracy scores indicate that manually written materials had the highest accuracy score (median=6, 25th to 75th percentile=5-6). Comparing the accuracy scores among the 3 sets of PEMs (ChatGPT: median=5, 25th to 75th percentile=4-5; DeepSeek: median=5, 25th to 75th percentile=4-6), the intergroup difference was statistically significant (Hedges *g*=7.27, *P*=.03; *δ*=0.02, 95% CI –0.51 to 0.54; *z*-score=–0.06, *P*=.96). However, after Bonferroni correction for multiple comparisons, the adjusted significance showed no statistically significant differences between any pairwise comparisons. For the comparison between ChatGPT and DeepSeek, the effect size was negligible, indicating no significant difference.

Among the 8 expert reviewers, 4 noted that the accuracy of the materials generated by ChatGPT-4o and DeepSeek R1 had issues. Two experts did not specify specific details but described the issues as follows: “Materials 2 (generated by ChatGPT) and 3 (generated by DeepSeek) contain some outdated content and lack scientific rigor, suggesting that professionals should further review them” and “Materials 2 and 3 contain some imprecise educational content or are not updated according to the latest guidelines/expert consensus, making them somewhat outdated and deviating from current clinical practices.” The other 2 experts pointed out specific problems: “Material 2 has outdated information (eg, the recommended daily salt intake was updated from 6 g to 5 g, but the material still mentions 6 g)” and “In Material 3‐1, it states that ticagrelor should be used with aspirin for 12 months, but in Material 3‐2, it mentions that ticagrelor should be continued for 12‐18 months post-stent placement. The duration of ticagrelor use is unclear in the material, which may confuse patients.” The frequencies of errors mentioned by the experts were 0% (0/8) for the manually written materials, 38% (3/8) for ChatGPT, and 38% (3/8) for DeepSeek.

## Discussion

### Principal Findings

#### Manually Written Versus Generative AI

This study demonstrated that the time required to create PEMs for patients with CHD was 14 hours when completed manually, while the use of ChatGPT took 0.62 hours and DeepSeek took 0.78 hours. These results suggest that generative AI can significantly improve the efficiency of developing PEMs for patients with CHD. By using structured prompts and leveraging contextual learning capabilities, generative AI can quickly process complex information and generate structured content [38]. This provides an unmatched level of work efficiency compared with manual methods and helps overcome the challenge that material developers often face when they are unfamiliar with specialized medical knowledge related to disease prevention and treatment. Additionally, the powerful text generation capabilities of generative AI allow health care professionals to produce more PEMs in the same amount of time as manual development, potentially addressing the scarcity of PEMs in primary health care settings [39,40].

In terms of readability, from the perspective of the 4 core language feature indicators, generative AI is more stable in its readability output compared with manually written materials, which is a significant advantage. Generative AI tends to use fewer personal pronouns than manually written materials, which can improve the coherence of the text and the clarity of references. The content word frequency in generative AI was lower than in manually written materials, indicating that its content words are more varied, leading to greater cognitive load. This suggests that, by modifying the prompts, generative AI can be instructed to increase content word frequency to reduce the cognitive load for patients and their families, thereby improving readability.

This study found that both manually written and AI-generated PEMs achieved a Chinese elementary school reading level in terms of the proportion of simple sentences and the number of personal pronouns. However, the frequency of difficult words and content word frequency corresponded to a high school reading level, which did not meet the requirement that PEMs should be written at the 6th-grade reading level [41]. This aligns with the findings of several studies [42-44] that showed that both AI-generated and manually written PEMs do not meet the recommended 6th-grade reading level.

This study shows that both manually written and AI-generated PEMs have good understandability and actionability, with comparable quality. However, generative AI is more stable overall than manually written materials. The results of this study, consistent with several others [44,45], demonstrate that the understandability and actionability of AI-generated PEMs are no different from those written by humans, indicating that generative AI is equally suitable for generating Chinese PEMs for CHD. This capability of generative AI depends not only on its large database and powerful computational power but also on the structured prompts [9] provided to it, which require converting complex medical information into easily understandable language. This suggests that, when developing PEMs, in addition to health care or health education professionals, experts in AI generation technology or training for health care professionals in AI generation technology are also needed. Furthermore, generative AI has a powerful self-learning capability, which can be enhanced by providing high-quality PEMs [46,47] to train the model, thereby improving the quality of output materials.

Although the statistical results showed no significant difference in the accuracy of the 3 sets of PEMs, this finding may indicate that there is no essential difference in accuracy between AI-generated materials and manually written materials, or it may be due to the small sample size of only 8 sets of data per method, leading to limited statistical power. It is also possible that the Bonferroni correction weakened the original significant differences due to its conservativeness. Despite the lack of significant statistical differences, the accuracy of content generated by generative AI still needs to be carefully evaluated. Of the experts consulted in this study, 4 raised concerns about the accuracy of AI-generated materials. For example, 1 expert explicitly pointed out: “Material 2 does not use the latest standard.” A study [48] indicated that 30% of pathology references generated by ChatGPT were incorrect, including inaccuracies and nonexistent references. Another review [19] mentioned that large models might confidently output incorrect or nonexistent answers. The “hallucinations” and bias issues of generative AI are real, and erroneous information could potentially harm patients, highlighting the necessity for strict review by health care professionals before using AI-generated PEMs in patient education to ensure the accuracy of the content.

Beyond accuracy, the use of AI in health care also carries inherent risks. A review [49] found that bias and lack of transparency are the 2 main risks of medical AI. This finding is particularly important for health care professionals planning to use generative AI to generate PEMs, as essential content that should be used to reduce bias might be omitted in the pursuit of readability. This further emphasizes the need for clinical review of content generated by generative AI.

#### ChatGPT Versus DeepSeek

This study shows that both generative AI tools used a small number of personal pronouns to enhance readability. DeepSeek had a higher proportion of simple sentences with greater variation than ChatGPT, which aligns with the findings of Zhou et al [50]. However, both models’ use of personal pronouns and the proportion of simple sentences reached the Chinese elementary school reading level. DeepSeek’s difficult words were more concentrated and stable compared with those generated by ChatGPT, but the difference between the 2 models was highly uncertain. DeepSeek had a significantly higher content word frequency than ChatGPT, although the PEMs from both models still fell within the high school reading level for these 2 aspects. A study [17] on generative AI’s ability to improve the readability of human-written PEMs showed that, although multiple generative AI tools improved the readability of PEMs, the generated content still generally exceeded the target requirements. Future research on generative AI specifically trained on medical data or fine-tuned for this task is worth exploring, particularly for reducing difficult words and increasing content word frequency to improve the overall readability of AI-generated materials.

Although the overall differences between the two generative AI tools in terms of understandability and actionability were not significant, ChatGPT was more stable in understandability, while DeepSeek was more stable in actionability. This suggests that, when using these 2 generative AIs to generate PEMs, more descriptive instructions can be provided to enhance their stability.

Accuracy is a common issue for both generative AI tools. In this study, experts pointed out that the number of errors in both models was the same. Two experts identified specific errors related to numerical information or issues where numerical information caused confusion for patients. This suggests that accuracy issues related to numerical information may be a common type of error for AI systems. These errors could stem from biases in the data used to train the AI system [49] or from intentional or unintentional manipulation of the data by the AI [51]. This highlights the importance of health care professionals maintaining a high sensitivity to the accuracy of numerical information when reviewing AI-generated PEMs.

DeepSeek R1 is a reasoning model [21]. After each prompt input, the model takes 7 seconds to 15 seconds to process and generate content. Although the initial content generated is logically coherent, it may include some areas where the content is overly specialized. Further optimization of the prompts, including additional rounds of interactions, is required to refine the content. ChatGPT is not a reasoning model and does not require thinking time, which explains why using DeepSeek takes slightly longer. At the same time, this study quantified the subtle differences in time consumption between the two, providing reference for health care professionals on how to choose the appropriate model.

### Limitations

This study has several limitations. First, only 3 manually written sets of PEMs for CHD by a single hospital were evaluated, which is a major limitation of this study. Expanding the scope to include more hospitals or different disease types may yield different results. Second, the target users of PEMs are patients and caregivers, but in this study, only health care professionals conducted the evaluations, which may introduce medical knowledge bias. Future research could involve evaluations by target users after strict assessment of accuracy by health care professionals. Additionally, the calculation of PEMAT-P scores was performed by researchers who were aware of the group assignments, which could introduce subjective bias. Third, generative AI tools are rapidly evolving, and future versions may perform better. Fourth, although a multidimensional evaluation was conducted, this study did not assess whether the AI-generated PEMs omitted any key information. Future research could explore the completeness of content generated by generative AI.

### Conclusions

The results of this study indicate that, compared with manually written materials, AI-generated PEMs for CHD demonstrate good outcomes in terms of time cost, understandability, and actionability, highlighting their potential for application. ChatGPT is more stable in understandability, while DeepSeek is more stable in actionability, suggesting that stability can be improved through more descriptive instructions. In terms of readability, although there are some differences between the 2 generative AI tools and between generative AI and manually written materials, the proportion of simple sentences and the number of personal pronouns in all 3 groups aligned with the reading level of Chinese elementary school students, while the difficulty of words and content word frequency corresponded to a high school reading level. The readability of AI-generated PEMs can be improved with more precise prompts.

In the future, generative AI models specifically trained on medical data or fine-tuned for this task could be developed to enhance the overall readability of AI-generated materials. Although there are certain flaws in accuracy, it is crucial to ensure that AI-generated PEMs for CHD undergo strict review by health care professionals to avoid outdated, incorrect, or nonexistent information being applied in clinical practice. In health care contexts, where information can impact human health and lives, it is essential to ensure that generative AI systems adhere to ethical standards and produce results that align with ethical considerations.

## Supplementary material

10.2196/78816 Multimedia Appendix 1 Prompt.

## Abbreviations

AI: artificial intelligence
CHD: coronary heart disease
CRIE: Chinese Readability Indicator and Evaluator
PEM: patient education material
PEMAT-P: Patient Education Materials Assessment Tool for Printable Materials
